# How experts’ own inconsistency relates to their confidence and between-expert disagreement

**DOI:** 10.1038/s41598-022-12847-5

**Published:** 2022-06-03

**Authors:** Aleksandra Litvinova, Ralf H. J. M. Kurvers, Ralph Hertwig, Stefan M. Herzog

**Affiliations:** Center for Adaptive Rationality, Max Planck Institute for Human Development, 14195 Berlin, Germany

**Keywords:** Psychology, Computational models

## Abstract

People routinely rely on experts’ advice to guide their decisions. However, experts are known to make inconsistent judgments when judging the same case twice. Previous research on expert inconsistency has largely focused on individual or situational factors; here we focus directly on the cases themselves. First, using a theoretical model, we study how within-expert inconsistency and confidence are related to how strongly experts agree on a case. Second, we empirically test the model’s predictions in two real-world datasets with a diagnostic ground truth from follow-up research: diagnosticians rating the same mammograms or images of the lower spine twice. Our modeling and empirical analyses converge on the same novel results: The more experts disagree in their initial decisions about a case (i.e., as consensus decreases), the less confident individual experts are in their initial decision—despite not knowing the level of consensus—and the more likely they are to judge that same case differently when facing it again months later, regardless of whether the expert consensus is correct. Our results suggest the following advice when faced with two conflicting decisions from a single expert: In the absence of more predictive cues, choose the more confident decision.

## Introduction

Experts often change their minds, sometimes with profound consequences. For example, a physician might initially classify a mass in a mammogram image as cancerous, but later—when re-inspecting the image—change their mind and classify it as benign. Which diagnosis should the patient rely on? Within-person inconsistency in expert judgments has been observed across many domains, including medicine^1–3^, clinical psychology^4^, neuropsychology^5^, forensics^6^, finance and management^7^, agriculture^8^, and weather forecasting^9^. Understanding the roots of within-expert inconsistency is crucial, as such inconsistency not only creates uncertainty for advice seekers about the right course of action, but may also erode societal trust in experts. Here we address two research questions: When do experts—in the absence of any new information—change their decisions? And, in the absence of more predictive cues, on which of the two decisions should experts or advice seekers rely on?

Most studies investigating within-person inconsistency in judgment and decision making have focused either on processes within the individual, such as probabilistic sampling of information^10,11^, a change of mind as revealed by post-decisional confidence pointing to the opposite decision^12,13^, and hierarchical hypothesis testing^14^, or on situational factors such as time pressure^15^. These and most previous studies have focused primarily on non-experts. Furthermore, how the cases themselves affect inconsistency in a person’s judgments has received comparatively little attention^4,16^.

To the best of our knowledge, we here present the first comprehensive investigation of the interplay between an expert’s confidence, consistency, and how clearly the information in a case points to one or the other decision (as indicated by how strongly experts agree on the case). We proceed in two steps: We start by using a theoretical model^17^ to investigate how within-person inconsistency and confidence in two-alternative forced-choice tasks are related to how clearly the information in a case points to either the correct or the incorrect decision (Study 1). We do this by relating an expert’s within-person consistency (also known as “intrarater agreement”) to the agreement among a population of experts (also known as “interrater agreement”). Next, we empirically test the model’s predictions in two real-world datasets with a diagnostic ground truth from follow-up research (Study 2): diagnosticians rating the same mammograms^18^ or images of the lower spine^19^ twice.

## Results

### Study 1: a theoretical model linking experts’ inconsistency, experts’ confidence, the agreement among cues in a case, and the agreement among experts for that case

A fundamental process assumed by many—but not all—models of cognition, judgment, and decision making is that individuals sample evidence from their environment or memory when making a decision^10,11,17,20^. This sampled evidence determines both the decision and the confidence in that decision^17,20,21^. A common assumption in such models is that an individual samples several pieces of evidence (“cues”) and selects the option for which there is stronger evidence. The more clearly the evidence points to that option, the more confident the individual will be in the accuracy of their decision. In this view, making a second decision about the same case at a later time point is equivalent to drawing a second sample of evidence—assuming that the individual does not know that they are judging the same case again or forgot their first decision. Because the sampling process is probabilistic, the evidence in the second sample may differ from that in the first sample and hence may lead to a different decision (e.g., “cancer” vs. “no cancer”) and associated level of confidence.

But how does inconsistency in repeated judgments relate to confidence and, in turn, how does confidence relate to how clearly the cues point to one or the other option (i.e., agreement among cues)? To investigate this question conceptually, we used the self-consistency model (SCM)^17^. It embodies the assumptions outlined above and allows us to derive qualitative predictions about the relationship between an expert’s inconsistency, confidence, and a case’s agreement among cues. We used the SCM because it permits for a straightforward illustration of the important concepts common to many models of judgment and decision making. Importantly, as we show later in this section, relaxing several of the assumptions of this basic model would not change the qualitative nature of the predictions. That is, the insights we present, based on the assumptions of the SCM, are representative of a much wider and empirically more realistic set of assumptions. Furthermore, in the “Discussion” section we argue that qualitatively similar predictions are also expected to emerge from other, more fine-grained models of judgment and decision making, such as evidence accumulation models^20,22^.

The SCM assumes that a decision maker facing a two-alternative choice task samples a fixed, odd number *n* of pieces of evidence (“cues”) from memory or the environment and chooses the option favored by more cues (i.e., decides between two options using majority voting among cues). Given a probability *p* of sampling a cue that indicates the correct option (say, “cancer”) and assuming that cues are sampled independently, the probability *P* of making a correct decision thus follows from the binomial distribution

1 $$\begin{aligned} P(p,n) = \sum \limits _{h = m}^n {\left( {\begin{array}{*{20}{l}} n\\ h \end{array}} \right) \cdot {p^h}{{(1 - p)}^{n - h}}}, \end{aligned}$$

where $m = \frac{{n + 1}}{2}$ (i.e., the minimum number of cues necessary to decide in favor of the correct option). Note that this model permits for so-called “wicked” cases, that is, cases in which the cues tend to point to the wrong option ($p < 0.5$)^23,24^. In various domains, including the two datasets analyzed in this study, a considerable subset of cases belong to this class. In contrast, in “kind” cases the cues tend to point, on average, to the correct option ($p > 0.5$) and thus the majority opinion tends to be correct^23,24^. Importantly, for most—if not all—domains one would typically expect more kind than wicked cases (“kind environments”), reflecting the assumption that the population of decision makers possesses at least some skill (i.e., their decision strategies show at least a minimal fit with the statistical structure of the cues in the environment). Situations in which there are more wicked than kind cases (“wicked environments”) are unrealistic because they imply that decision makers perform worse than chance (i.e., exhibit negative skill).

This basic decision model makes two assumptions that are unlikely to hold in practice. First, it assumes that all cues are equally informative (i.e., $p_i = p$ for all cues *i*), and, second, that the cues are independent (or more specifically, that the binomial sampling process is independent and identically distributed). For example, in medical diagnostics, some diagnostic cues will be more informative than others, and certain cues will tend to co-occur for a particular disease. Relaxing these two assumptions would change the exact functional form between the probability of making a correct decision, *P*, and the two other variables defined below (confidence and inconsistency). However, as we also show below, the qualitative predictions  we derive remain intact for a broad range of alternative assumptions.

The SCM further stipulates that confidence $\widehat{C}$ in a decision increases with the proportion of cues pointing to the chosen option ($\widehat{p}$ for correct decisions and $1 - \widehat{p}$ for incorrect decisions). In particular, the SCM postulates that confidence $\widehat{C}$ is the complement of the sample standard deviation of $\widehat{p}$:

2 $$\begin{aligned} \widehat{C} = 1 - \sqrt{\widehat{p}\left( 1 - \widehat{p} \right) }. \end{aligned}$$

SCM’s definition of confidence^17^ assumes that people’s confidence increases faster than linearly with the proportion of cues pointing to the chosen option. However, the qualitative predictions we derive below depend only on the assumption that confidence is monotonically increasing with the proportion of cues pointing to the chosen option. That is, $\widehat{C} \propto max \left( 1 - \widehat{p}, \widehat{p}\right)$. Therefore, other possible and justifiable definitions of confidence would result in the same qualitative predictions (e.g., using precision: $\frac{1}{var(\widehat{p})} = \frac{1}{\widehat{p}\left( {1 - \widehat{p}} \right) }$).

**Figure 1:**
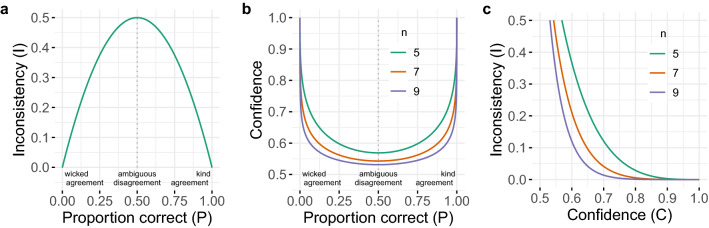
Predictions of the self-consistency model on the relations between the proportion of individuals who make a correct diagnosis (*P*), inconsistency (*I*; probability of making two inconsistent decisions), and confidence (*C*) for three values of *n* (number of sampled cues; color coded in **(b,c)**). **(a)** Inconsistency as a function of the proportion of individuals making a correct decision. Note that this relationship does not covary with *n* (see Eq. (3)). **(b)** Confidence as a function of the proportion of individuals making a correct decision. **(c)** Inconsistency as a function of confidence. Note that the probability *p* with which a cue points to the correct option does not appear because, given any fixed number *n* of sampled cues, *p* and *P* are monotonically related (see Eq. (1)); thus the curves in **(a,b)** would not yield qualitatively different insights if *p* (instead of *P*) were shown on the x-axes.

Next, we analytically derive the relations between inconsistency, confidence, and the proportion of cues pointing to the chosen option. The probability *I* of making two decisions that are inconsistent is

3 $$\begin{aligned} I = P(1 - P) + (1 - P)P = 2P(1 - P), \end{aligned}$$

which is maximal ($I = 0.5$) for choices at chance level ($P = 0.5$) and, by extension, for cases that are maximally ambiguous ($p = 0.5$)—that is, when every sampled cue is equally likely to either point to the correct or the incorrect option (Fig. 1a). Conversely, inconsistency is minimal ($I = 0$) for perfectly correct ($P = 1$) and “perfectly” incorrect ($P = 0$) decisions—that is, when every sampled cue points either to the correct option ($p = 1$; perfectly “kind” cases) or to the incorrect option ($p = 0$; perfectly “wicked” cases^23,24^). Thus, in the SCM, within-expert inconsistency increases the closer a case’s *p* is to a fair coin flip (i.e., a inconsistency is a monotonically decreasing function of $|p - 0.5|$).

The SCM provides a simple, elegant link between the consistency of a single person’s repeated decisions for a case and between-person agreement on that same case. For simplicity, let us assume that all experts sample the same number of cues (i.e., share a common *n*) and that for any particular case all experts have the same probability *p* of sampling a cue that points to the correct option. Although *p* is not directly observable, according to the SCM, the expected proportion of correct decisions $E[P_i(p_i)]$ for case *i* among a population of identical experts is monotonically related to $p_i$. Empirically, the sample proportion of correct decisions among experts for case *i*, $\widehat{P_i}$, can be used as a proxy for ordering cases according to their $p_i$. Because Eq. (1) applies to majority voting over either cues or individuals, we can use Condorcet’s jury theorem^25,26^ to gain insights into how $P_i$ and $p_i$ relate for $n \ge 3$. For example, for $p_i> 0.5 \rightarrow P_i > p_i$; conversely, for $p_i< 0.5 \rightarrow P_i < p_i$. Thus, if we assume that experts sample three or more cues, $P_i$ will be a more extreme version of $p_i$. Particularly, for any $n \ge 3$, $P_i$ and $p_i$ are identically ordered across a set of cases. Furthermore, research on majority voting^26–28^ has shown that the gist of Condorcet’s jury theorem holds even when the standard assumptions are violated. For example, $p_i> 0.5 \rightarrow P_i > p_i$ and $p_i< 0.5 \rightarrow P_i < p_i$ hold even if the cues differ in their probability of pointing to the correct option as long as their $p_i$ are symmetrically distributed around $\widehat{p}_i$^26^ or if the cues are interdependent, as long as their intercorrelations are not extreme^27^. In sum, we can use the disagreement among experts (i.e., how close $\widehat{P_i}$ is to 0.5) as an indicator of the disagreement among the cues for the case at hand (i.e., how close $p_i$ is to 0.5, that is, $|p - 0.5|$).

Because the sample proportion of cues pointing to the correct option, $\widehat{p}$, equals *E*(*p*), confidence is highest for $p = 1$ and $p = 0$ ($\widehat{C} = 1$) and lowest for $p = 0.5$ ($\widehat{C} = 0.5$; see Eq. (2), Fig. 1b)—mirroring the results for an individual expert’s inconsistency (see Eq. (3), Fig. 1a). Given that inconsistency *I* increases and confidence $\widehat{C}$ decreases with increasing case ambiguity (i.e., as *p* gets closer to 0.5), it follows that confidence and inconsistency are negatively related (Fig. 1c). Note that these relations hold for any definition of confidence in which confidence monotonically increases with the proportion of cues pointing to the same decision.

But which decision should a person confronted with two inconsistent, conflicting decisions rely on? According to the *maximum-confidence slating* (MCS) algorithm^24^ (henceforth “confidence rule”), they should adopt the more confident decision^29,30^. The SCM predicts that confidence will be positively correlated with the probability of making a correct decision for $p > 0.5$, but negatively correlated for $p < 0.5$ (Fig. 1b). More specifically, Eqs. (1) and (2) show that, for $p > 0.5$, any level of confidence is more likely to be observed under the correct than the incorrect decision (and vice versa for $p < 0.5$). To see why, consider that in Eq. (1), $\widehat{p} = \frac{h}{n}$. When $p > 0.5$, it follows that $p^h > (1 - p)^{n - h}$ and thus the event that a majority of cues point to the correct decision (*h*) is more likely than the event that the same-sized majority of cues point to the incorrect decision ($n - h$). The opposite is the case when $p < 0.5$. Adding the assumption that there are more kind than wicked cases in a domain (i.e., the environment is overall kind) implies that, everything else being equal, confidence positively predicts accurate decisions. Thus, confidence’s predictive ability depends on the distribution of the cases’ $p_i$ in a domain.

In sum, the SCM predicts:

1. The more experts disagree in their initial decisions about a case (i.e., as case consensus decreases), the more likely it is that an individual expert will judge the case differently upon seeing it again—despite their lack of knowledge about the level of agreement or disagreement among all experts (Fig. 1a).
2. The more experts disagree in their initial decisions about a case (i.e., as case consensus decreases), the less confident an individual expert will be in their initial decision—despite their lack of knowledge about the level of agreement or disagreement among all experts (Fig. 1b).
3. The less confident an individual expert is in their initial decision about a case, the more likely it is that they will judge the case differently when facing it again (Fig. 1c).
4. If an expert makes two conflicting decisions, using the confidence rule (i.e., selecting the more confident decision) improves accuracy for kind cases but worsens it for wicked cases when compared to first and second decisions.

Note that predictions 1–3 do not depend on whether the experts’ consensus opinion is correct or not.

### Study 2: empirical test

The theoretical predictions derived in Study 1 depend on the SCM’s assumptions about an expert’s decision process and our additional assumption that all experts are identical in terms of SCM’s parameters. Specifically, the model assumes that all experts sample the same number *n* of cues and have, for a given case, the same probability *p* of independently sampling a cue that points to the correct option. As discussed in Study 1, these assumptions are unlikely to hold for actual expert decisions; Study 2 therefore tests the model’s qualitative predictions by re-analysing two real-world expert datasets: diagnosticians rating mammograms^18^ and X-rays of the lower spine^19^ twice (see “Methods” section for details on these two datasets).

**Figure 2:**
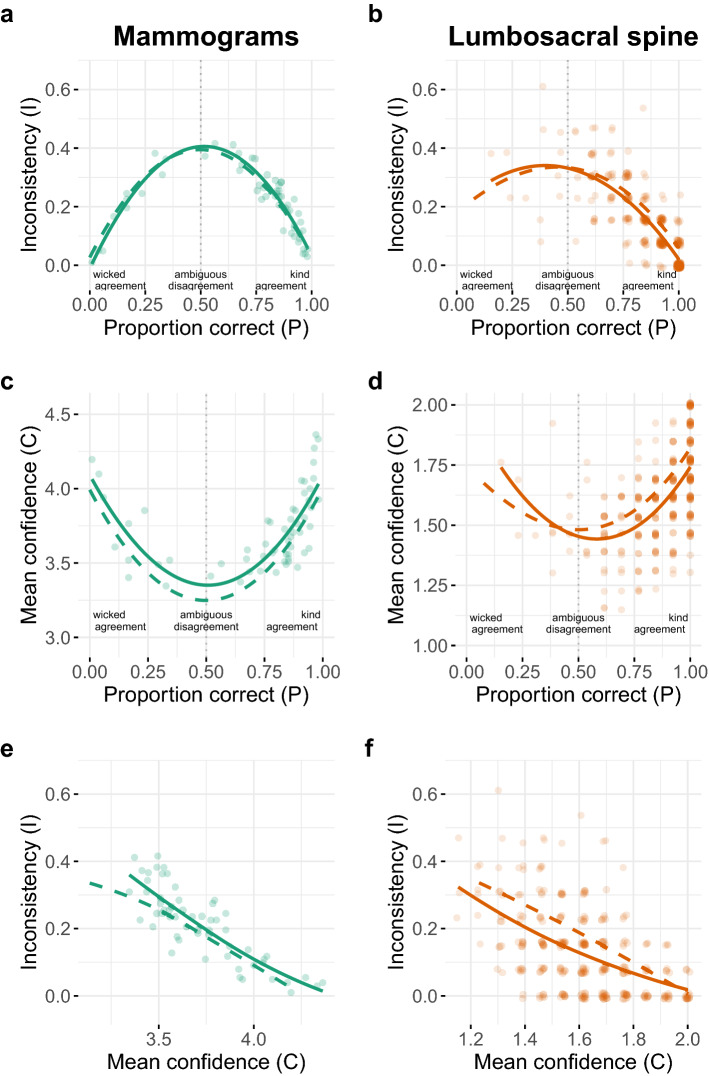
Empirical results on the relationship between the proportion of experts who made a correct diagnosis ($\widehat{P}$), inconsistency ($\widehat{I}$; probability of making an inconsistent diagnosis), and mean confidence ($\widehat{C}$) in the two datasets. **(a,b)** Inconsistency per case (i.e., proportion of experts who gave two different diagnoses) as a function of the proportion of experts who made a correct initial diagnosis for that case. **(c,d)** Mean confidence in the initial diagnosis per case as a function of the proportion of experts who made a correct diagnosis for that case. In the mammography dataset **(c)**, confidence was elicited on a 5-point rating scale (1: “not at all confident”, 2: “not very confident”, 3: “neutral”, 4: “confident”, 5: “very confident”). In the spine dataset **(d)**, confidence was elicited on a 2-point scale (1: low confidence, 2: high confidence). **(e,f)** Inconsistency per case as a function of its mean confidence. Each dot represents one case and its coordinates represent $\widehat{P}$ and $\widehat{C}$ from initial diagnoses (i.e., from the first rating session in the respective dataset); the solid curves are LOESS smooths across those points. The dashed curves show the smooths when using $\widehat{P}$ and $\widehat{C}$ from the second diagnoses (i.e., from the second rating session in the respective dataset); to avoid overplotting, the corresponding dots for the individual cases are not shown. **(b,d,f)** Employ jittering to avoid overplotting.

In the first dataset, 102 radiologists rated up to 109 mammograms twice, with an interval ranging between 3 and 9 months^18^. On average, the experts changed their diagnoses in about one in five cases (median proportion: 21%, interquartile range, IQR: 0.14–0.28). In the second dataset, 13 physicians rated 300 images of the lumbosacral spine twice, with a delay of 3 months^19^. The experts changed their diagnoses in about one in eight cases (13%, IQR: 0.09–0.15).

In both datasets, practitioners indicated their confidence in the given diagnosis. Importantly, in both datasets there is a diagnostic ground truth based on follow-up research (see “Methods” section for details), which we use to score the accuracy of a diagnosis. Experts’ average performance was better than chance in both datasets, but substantially better in the spine dataset (see Supplementary Fig. S1). Furthermore, the proportion of wicked cases (i.e., the proportion of cases where the majority of experts gave the incorrect diagnosis) was lower in the spine dataset. As a consequence, predictions 1, 2, and 4 can be assessed with higher precision for kind than for wicked cases—especially in the spine dataset.

For statistical inference, we ran a series of Bayesian mixed-level regression models, which included group-level intercepts for individuals and cases (“random intercepts”; see Supplementary Table S1 and Supplementary Method for details).

Prediction 1: The more experts disagree in their initial decisions about a case (i.e., as case consensus decreases), the more likely it is that an individual expert will judge the case differently upon seeing it again—despite their lack of knowledge about the level of agreement or disagreement among all experts.

Figure 2a,b shows that, as predicted, the more disagreement there was in experts’ initial diagnoses, the more likely it was that an individual expert gave the opposite diagnosis when judging the same case again months later—irrespective of whether the between-expert majority opinion was correct and despite not knowing the level of agreement or disagreement among all experts. These results were particularly clear in the mammography dataset (Fig. 2a). In the spine dataset, the predicted pattern clearly emerged for kind cases (Fig. 2b); the results for the wicked items are also consistent with the prediction, but less conclusive (Fig. 2b). Regression model M2 (Supplementary Table S1) shows clear evidence for a negative quadratic term in both datasets, supporting the visual impression from Fig. 2a,b. Moreover, the regression model M1 (Supplementary Table S1) shows that—prior to accounting for a case’s level of agreement or disagreement among all experts—the cases differed much more strongly in how inconsistently they were diagnosed than the experts differed in how inconsistently they diagnosed those same cases. This finding further strengthens the relevance of a case’s ambiguity in explaining variation in within-expert inconsistency.

Prediction 2: The more experts disagree in their initial decisions about a case (i.e., as case consensus decreases), the less confident an individual expert will be in their initial decision—despite their lack of knowledge about the level of agreement or disagreement among all experts.

Figure 2c,d shows that, as predicted, the more experts disagreed in their initial diagnoses, the less confident they were in their initial diagnoses—irrespective of whether the between-expert majority opinion was correct and despite not knowing the level of agreement or disagreement among all experts. Regression model M4 (Supplementary Table S1) shows a clearly positive quadratic term in both datasets, supporting the visual impression from Figure 2c,d.

Prediction 3: The less confident an individual expert is in their initial decision about a case, the more likely it is that they will judge the case differently when facing it again.

Figure 2e,f shows that, again as predicted, the less confident an expert was when judging a case, the more likely they were to arrive at the opposite diagnosis when judging that same case again months later. Regression model M5 (Supplementary Table S1) shows a clearly negative linear term in both datasets, substantiating the visual impression from Fig. 2e,f.

Prediction 4: If an expert makes two conflicting decisions, using the confidence rule (i.e., selecting the more confident decision) improves accuracy for kind cases but worsens it for wicked cases when compared to first and second decisions.

**Figure 3:**
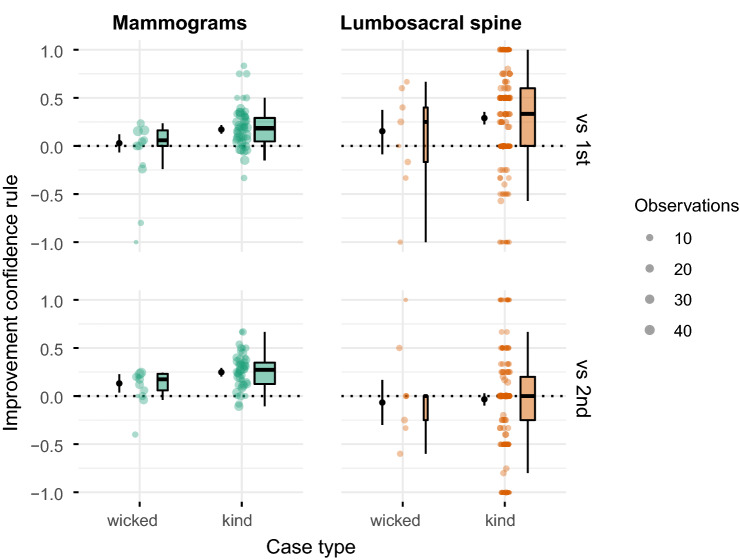
Empirical results comparing the accuracy of the confidence rule to the accuracy of first (upper panel) and second (lower panel) diagnoses for cases where experts were inconsistent, separately for wicked and kind cases and the two datasets (mammography and lumbosacral spine). Positive (negative) decimals on the y-axes show the extent to which the confidence rule increased (decreased) performance compared to the respective other strategy. Cases are shown as horizontally jittered dots; the size of a dot indicates the number of experts who provided different diagnoses for that case (see the legend labelled “Observations”). The distributions are summarized by boxplots (to the right of the dots); the boxplots consider the number of observations per case (weighted boxplots) and their width is proportional to the square root of the number of cases in the respective distribution. The point and line range to the left of the dots indicates the median and 95% credible interval of the posterior distribution of the expected average improvement (according to model M7; see Supplementary Table S1).

Focusing only on the cases where an expert provided opposing diagnoses, we found that, relative to the initial diagnosis, using the confidence rule (i.e., selecting the more confident diagnosis) improved accuracy for kind items (Fig. 3, upper panels). For wicked items (i.e., cases where the majority of initial diagnoses were incorrect), the confidence rule performed at par with the initial diagnosis and thus our prediction was not corroborated.

**Figure 4:**
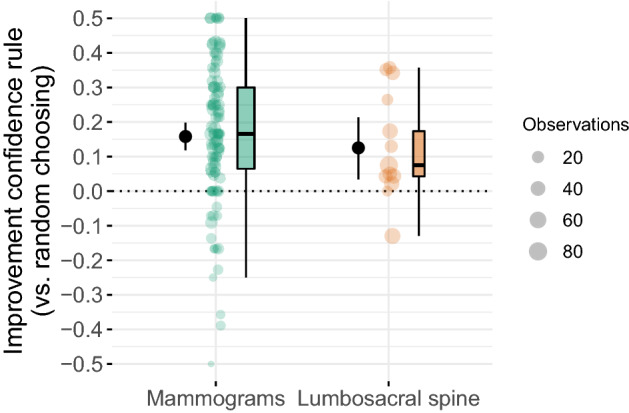
Empirical results comparing the accuracy of the confidence rule to the accuracy of choosing randomly between first and second diagnoses for cases where experts were inconsistent, separately for the two datasets (mammography and lumbosacral spine). The y-axis shows by how much the confidence rule increased accuracy compared to randomly choosing; negative values indicate by how much the confidence rule decreased performance. Experts are shown as horizontally jittered dots; the size of a dot indicates the number of cases for which that expert provided conflicting diagnoses (see the legend labelled “Observations”). The distributions are summarized by boxplots (to the right of the dots); the boxplots consider the number of observations per case (weighted boxplots) and their width is proportional to the square root of the number of experts in the respective distribution. The point and line range to the left of the dots indicate the point estimate of a Bayesian one-sample t-test (median of the posterior distribution of the distribution’s mean, as well as the corresponding 95% credible interval; using the standard “ultrawide” prior scale)^31^. Using the effect size $\delta$ (the difference of the mean to zero, divided by the standard deviation)^31^, the increase amounts to 0.76 (median of the posterior distribution of $\delta$; 95% credible interval, CI 0.55–0.98) in the mammography dataset and to 0.81 (CI 0.19–1.46) in the lumbosacral spine dataset.

Comparing the confidence rule to the experts’ second judgement yielded largely similar results. In the mammography dataset, the confidence rule increased performance for kind cases, and performed at par for wicked cases (Fig. 3, lower-left panel). In the spine dataset, however, the confidence rule performed at par with the second diagnosis for both kind and wicked cases (Fig. 3, lower-right panel); this presumably happened because second diagnoses in the spine dataset were substantially more accurate than first diagnoses (see Supplementary Fig. S1b). In contrast, in the mammography dataset, the accuracy level was comparable across first and second diagnoses (S1a). Regression model M7 supports these observations (see Supplementary Table S1). Importantly, summarizing across all cases, the confidence rule clearly outperformed the strategy of randomly choosing between the first and second diagnosis (Fig. 4).

## Discussion

When do experts change their mind? Previous research on within-person inconsistency has focused largely on individual^10,11^ or situational factors^15^. In contrast, factors that relate to the task in question and that may contribute to experts’ consistency or lack thereof have rarely been studied (e.g., consistency decreases as a task becomes less predictable^16,32^). We therefore focused directly on the cases themselves. First, using the Self-consistency model (SCM)^17^, we studied how inconsistency and confidence in two-alternative forced-choice tasks are related to how clearly the information in a case points to either the correct or the incorrect option (agreement among the cues for a case, proxied by the level of agreement among the population of experts’ initial diagnoses). Next, we found support for three of the model’s four key predictions in two real-world datasets—diagnosticians rating the same mammograms or images of the lower spine twice. We found that the more experts’ initial diagnoses of a case differed, the more likely individual experts were to change their diagnosis months later (prediction 1), and the less confident they were in their initial diagnosis (prediction 2)—irrespective of whether the expert consensus (i.e., majority diagnosis) for that case was correct and despite experts being unaware of the level of agreement or disagreement among all experts. Consequently, the more confident an expert was in their initial diagnosis, the less likely they were to change their diagnosis when judging the same case again months later (prediction 3). This held irrespective of whether the consensus diagnosis for that case was correct and despite experts not knowing the level of agreement or disagreement. Taken together, these first three results imply that a highly confident or consistent diagnosis is, first and foremost, an indicator for the level of agreement among experts. It can only be an indicator of accuracy when most cases in the domain of interest are kind (i.e., for the majority of cases, the experts tend to give the correct response).

When an expert’s two diagnoses were inconsistent, the confidence rule (i.e., selecting the more confident diagnosis) improved accuracy relative to keeping the initial diagnosis for kind cases; however, the opposite result predicted for wicked cases (prediction 4) was only partially corroborated. These mixed findings might result from systematic differences in accuracy and confidence judgments between first and second diagnoses, especially for the spine dataset (e.g., second spine diagnoses were more accurate and more confident than first diagnoses; see Supplementary Figs. S1, S2). Importantly, however, summarizing across all cases the confidence rule outperformed both first and second diagnoses in the mammography dataset and first, but not second, diagnoses in the spine dataset (see model M6 in Supplementary Table S1). Furthermore, the confidence rule clearly outperformed the strategy of randomly choosing between the first and second diagnosis (Fig. 4). This finding agrees with another study showing that applying the confidence rule to pathologists’ and laboratory professionals’ diagnoses of white blood cells improved accuracy, relative to randomly choosing between the two diagnoses^33^. Because decision makers cannot tell in advance whether a particular case is kind or wicked^34^, using the confidence rule has clear practical merit^35^. Our results suggest the following advice: In the absence of more predictive cues (and unless one suspects that experts perform below chance), rely on the more confident of an expert’s two conflicting decisions.

Our implementation of the SCM assumes that all experts sample the same number *n* of cues and have the same probability *p* of independently sampling a cue that points to the correct answer. As discussed earlier, these assumptions are unlikely to hold in practice. Yet, relaxing them does not qualitatively change any of the four predictions. It will affect the functional form with which the probability of a correct decision, *P*, depends on *p* (the probability of sampling a cue that points to the correct option) and *n* (the number of cues sampled) or how *p* influences confidence $\widehat{C}$, but the qualitative implications of the distinction between kind cases ($p > 0.5$) and wicked cases ($p < 0.5$) is expected to remain unchanged. Because SCM’s decision process amounts to majority voting among cues we can draw on insights from research on majority voting. For example, predictions 1–4 hold even if, within an expert, the cues differ in their probability of pointing to the correct option as long as either their $p_i$ are symmetrically distributed or the cues are interdependent and their intercorrelations are not extreme^27^. As another example, under very general conditions, as the number of cues retrieved, *n*, increases, the probability of a correct decision, *P*, will increase for kind cases ($p > 0.5$) and decrease for wicked cases ($p < 0.5$)^26–28^. As a consequence, all else being equal, consistency should increase as more cues are retrieved; variations in how strongly the cues within a case agree with each other will be most pronounced for small *n*s, whereas for large *n*s all cases will be clearly diagnosed either correctly or incorrectly (except for cases with *p* close to 0.5). Furthermore, assuming that experts sample different numbers of cues implies that, for the same case, experts with larger *n*s will be more consistent than experts with smaller *n*s. Importantly, experts with larger *n*s will only be more accurate for kind cases; they will be less accurate for wicked cases because they are less likely to arrive at the correct decision by random chance (i.e., sampling error leading to a sample of cues pointing to the correct option despite $p < 0.5$). The SCM stipulates that confidence in a decision increases with the proportion of cues pointing to that option (see Eq. (2)). Any definition of confidence in which confidence increases monotonically with $|\widehat{p} - 0.5|$ will yield the same qualitative conclusions as the definition we relied on (see Eq. (2)).

Here we used the SCM as a basic model linking accuracy, confidence, consistency, and consensus because it allows for a straightforward illustration of those interrelations. However, we argue that a broad family of models make qualitatively similar predictions to those of the SCM. For example, in the diffusion decision model^22^, a prominent example from the family of evidence accumulation models, the agreement among cues within a case is reflected in the drift rate, which represents the average speed with which an expert accumulates evidence that stochastically drifts toward one of two decision boundaries (e.g., choice A vs. choice B, or cancer vs. no cancer). Everything else kept constant, a lower drift rate  implies more ambiguous cases, which are predicted to be associated with lower accuracy, longer response times, and lower confidence within experts^20,22^, as well as increasing disagreement among experts. Wicked cases correspond to situations where the mean drift rate points to the boundary representing the incorrect response. Notably, an increasingly wrong drift rate corresponds to increasingly less ambiguous and more wicked cases. These cases are predicted to be associated with even lower accuracy, but also with shorter response times and higher confidence within experts, as well as increasing agreement among experts on the incorrect decision—thus qualitatively mirroring the predictions from the SCM. More generally, any model that assumes or implies the following should make predictions qualitatively similar to those of the SCM: The more clearly the relevant information points to an option, the more likely a particular decision becomes and the more confidently it will be rendered. Arguably, these two relations are fundamental to many psychological and normative models of decision making; the key question is how a given model operationalizes the notion of how definitely information points to the chosen decision. However, certain decision strategies might not yield the same relationship between confidence, inconsistency, and case ambiguity (e.g., lexicographic rules, tallying strategies^36^, or exemplar-based strategies^37,38^). Furthermore, our empirical analyses are based on expert diagnoses of mammograms and X-rays of the lumbosacral spine and thus may not generalize to other expert domains (e.g., forensics or clinical psychology). The generality of our results should therefore be the subject of future research.

In the following, we discuss three contributions the present approach makes to research on within-expert inconsistency. First, in order to reduce inconsistency and thus improve accuracy, previous perspectives suggest using interventions that increase the reliability of information processing, such as reducing the amount of information presented^39^, decomposing a complex task into smaller ones^40^, or combining an individual’s repeated judgments^41–43^ or judgments from different individuals^44^. Our work suggests a complementary approach to improving accuracy in the face of unreliability—namely, encouraging experts to make a second assessment whenever they are not confident in their initial decision and to then apply the confidence rule across the two decisions. The rationale is twofold. One can expect that experts will perform better than chance and that the confidence rule will, therefore, improve accuracy relative to simply sticking to the initial decision. Also, there is little benefit in judging cases again that were initially diagnosed with high confidence; such decisions are unlikely to change and the confidence rule will therefore not change the final decision.

Second, previous accounts of expert inconsistency explicitly or implicitly assume that accuracy increases as the consistency of decisions increases^45^. In stark contrast to this assumption, our results show that this relationship is mirrored at chance level: For cases that experts tend to judge incorrectly, individual expert consistency increases the more experts agree on the incorrect diagnosis—despite the individual expert not knowing the level of agreement among all experts. Furthermore, our results show that confidence tracks consistency, but because confidence tracks the ambiguity of a case (or, equivalently, experts’ disagreement) and not accuracy per se^17^, the ability of confidence to predict accuracy and consistency strongly depends on the distribution of ambiguity across cases^29,43,46^. If there are only kind cases (i.e., cues tend to point to the correction option), confidence strongly predicts that a diagnosis is accurate and will not be changed. The more wicked cases there are, the more these relations dilute. In the extreme—and hopefully only hypothetical—case of a domain where experts, on average, tend to make wrong decisions, the relations reverse: Experts’ confidence is negatively related to accuracy, but still positively related to consistency—and being consistent in a wicked environment means confidently sticking to the wrong decision.

Third, previous accounts have focused on differences in consistency among experts or in different task conditions (e.g., time pressure). Our approach predicts that the cases themselves can differ markedly in terms of how consistently they are diagnosed by any expert. As our results have shown, these differences in consistency among cases can be even larger than those observed among experts and can be explained to a large degree by the extent to which experts disagree on a case.

## Methods

### Study 1

To facilitate the explanation of the Self-consistency model (SCM)^17^ we present its equations in the “Results” section. The code for Fig. 1 is available at https://osf.io/e7nk6/.

### Study 2

The code to analyze both datasets can be found at https://osf.io/e7nk6/. See the Data availability statement for information on how to obtain the two datasets.

#### Dataset 1: radiologists diagnosing mammograms

Dataset 1 consists of repeated judgments of the same mammograms. Here we re-analyze the data from a previous study that investigated the effect of time spent viewing and confidence on diagnostic accuracy in mammography screening^18^. On the topic of ethics approval the original paper^18^ noted:

This study was conducted with mammography registries (Carolina Mammography Registry, New Hampshire Mammography Network, New Mexico Mammography Project, Vermont Breast Cancer Surveillance System, and Group Health Cooperative in western Washington) associated with the National Cancer Institute-funded Breast Cancer Surveillance Consortium (BCSC). Data collected as part of this study were pooled at the BCSC Statistical Coordinating Center (SCC) in Seattle, WA, for analysis. Each registry and the SCC received institutional review board (IRB) approval for either active or passive consenting processes or a waiver of consent to enroll participants, link data, and perform analytic studies. All procedures are HIPAA compliant and all registries and the SCC have received a Federal Certificate of Confidentiality and other protection for the identities of the women, physicians, and facilities that are the subjects of this research. In addition, each registry and the SCC received IRB approval for all test set study activities.

Of the 469 radiologists invited to participate, 102 completed both phases of the study. The mammograms used were randomly selected from screening examinations of women aged 40–69 years. The correct diagnosis (cancerous or non-cancerous) for each mammogram was available from follow-up research. In phase 1, each radiologist was randomly assigned to one of four test sets of 109 mammograms. The radiologists were instructed to interpret the cases as they would in clinical practice. They were informed that the overall cancer rate in their test set was higher than that found in a screened population, but they were not informed of the specific prevalence of cancer cases. When viewing each case, radiologists were prompted to identify the most significant breast abnormality and to decide whether the patient should be recalled for additional workup. The decision to recall constituted a positive test result. Additionally, radiologists provided a confidence judgment for each assessment on a 5-point scale (1: “not at all confident”, 2: “not very confident”,  3: “neutral”, 4: “confident”, 5: “very confident”). Radiologists used a home or work computer or a laptop provided by the study to complete the task. After an interval ranging between 3 and 9 months, the same radiologists were invited to rate a second set of 110 mammograms, following an identical procedure. Unknown to the participants, a subset of the cases presented in this phase 2 were the same as in phase 1. Overall, 58 cases were rated twice by 55 radiologists; of those 58 cases, 46 were rated twice by another 47 radiologists, resulting in 5352 repeated ratings. All repeated mammograms were non-cancer cases (i.e., from women who were cancer-free for at least 2 years after the mammography). See^18^ for more details.

Across all repeated cases, the median accuracy (proportion correct) was 0.72 in the first phase and 0.68 in the second phase (see also Supplementary Fig. S1a). Experts’ first and second diagnoses were similarly confident (median within-expert mean confidence was 3.8 for first diagnoses and 3.7 for second diagnoses; see Supplementary Fig. S2a).

#### Dataset 2: physicians diagnosing X-rays of the lumbosacral spine

Dataset 2 consists of repeated judgments of X-rays of the lumbosacral spine. Here we re-analyze the data from a previous study that investigated the diagnostic accuracy of radiologists and chiropractors (total $N = 13$) reading lumbosacral radiographs^19^. The medical ethical committee of the Alkmaar hospital approved the study.

Five chiropractors, three chiropractic radiologists, and five medical radiologists participated in the study. Their professional experience ranged from 3 to 21 years. For the study, 300 X-rays of the lumbosacral spine of adult patients were selected from a hospital database. These consisted of 50 X-rays containing a “significant abnormality” (in which case immediate referral to a hospital is required) and 250 “normal” ones. X-rays with abnormalities were selected retrospectively based on an initial radiologic report. These radiographic findings were confirmed by a combination of other diagnostic imaging methods including magnetic resonance imaging (MRI) and computed tomography (CT). The selected X-rays overrepresented “significant abnormalities” (17 % of cases), including infections (*n* = 7), malignancies (*n* = 15), fractures (*n* = 8), inflammatory spondylitis (*n* = 6), and spondylolysis (*n* = 14). The set of X-rays was presented in a random order. For each X-ray, the physician evaluated whether a significant abnormality was present (yes vs. no) and gave a confidence rating on a 2-point scale (1: low confidence, 2: high confidence). Three months later, all participants assessed all 300 X-rays again, resulting in 3900 repeated assessments. See^19^ for more details.

Across all cases, the median accuracy (proportion correct) was 0.86 in the first session and 0.91 in the second session (see also Supplementary Fig. S1b). For six out of the 13 experts, their second diagnoses were more confident than their first ones; for two experts, their second diagnoses were less confident, and for five experts there was no reliable difference (see Supplementary Fig. S2b).

#### Statistical analyses

We ran a series of Bayesian mixed-level regression models^47^, which included group-level intercepts for individuals and cases (“random intercepts”; see Supplementary Information for detailed model descriptions and results). Note that each of the 300 X-rays in the spine dataset was rated by just 13 experts. Consequently, the estimates for both proportion correct $\widehat{P_i}$ and inconsistency $\widehat{I_i}$ are more noisy. In the mammography dataset, in contrast, up to 102 radiologists rated 58 distinct mammograms, allowing the characteristics of the cases to be estimated more reliably. To render our classification of cases as kind versus wicked more reliable, we defined—in both datasets—kind cases as $\widehat{P_i} > 0.6$ and wicked cases as $\widehat{P_i} < 0.4$. We thus excluded cases where $0.4 \le \widehat{P_i} \ge 0.6$ in model M7 (Supplementary Table S1); those cases were retained in all other analyses and figures (except Fig. 3).

## Supplementary Information

Supplementary Information.

## Data Availability

The spine dataset analyzed in this study is available on OSF (https://osf.io/e7nk6/). The mammography data that support the findings of this study are available from https://www.bcsc-research.org/contact. However, restrictions apply to the availability of these data, which were used under license for the current study, and so are not publicly available.
