# Supplementary material for: How experts’ own inconsistency relates to their confidence and between-expert disagreement
Source: Sci Rep. 2022 Jun 3;12:9273. doi: 10.1038/s41598-022-12847-5 (PMC9166728; doi:10.1038/s41598-022-12847-5)
Supplement: Supplementary file 1 — Supplementary Information. [file 41598_2022_12847_MOESM1_ESM.pdf]

Supplementary Information: How experts' own inconsistency relates to their confidence and between-expert disagreement

Aleksandra Litvinova*, Ralf H. J. M. Kurvers,
Ralph Hertwig, & Stefan M. Herzog*
Max Planck Institute for Human Development, Center for Adaptive Rationality

* contributed equally

Contents

Regression Analyses **1**
Methods 1
Results 3

Additional Results **5**

References **7**

Regression Analyses

Methods

We ran a series of Bayesian mixed-level regression models (see Supplementary Table S1) using the R package *brms* (version 2.16.3) and its default priors (Bürkner, 2017). The models all included group-level intercepts for individuals and cases (aka "random intercepts"). Four chains, each with 6,000 samples, were run. The first 2,000 samples were discarded as warm up. After thinning to reduce memory load (by a factor of 4), a total of 4,000 samples were obtained per model. The MCMC diagnostics did not indicate any problems. For the models' Stan code, MCMC diagnostics and the posterior distributions of the population-level parameters, see supplementary code and outputs at `https://osf.io/e7nk6/` in folder *output/models*.

The three models of inconsistency (M1, M2, and M4) are logistic regression models; their parameters thus indicate changes in log odds. The two models of confidence (M3 and M5) are linear models (i.e., identity link).

$(\widehat{P}-0.5)$ and $(\widehat{P}-0.5)^2$ in models M2 and M4 are the linear and quadratic polynomial contrasts of the 0.5-centered proportion of correct diagnoses per case; this means that the intercept in those models predicts the value of the dependent variable for a maximally ambiguous case ($\widehat{P} = 0.5$; because for $\widehat{P} = 0.5$, $(\widehat{P} - 0.5) = (\widehat{P} - 0.5)^2 = 0$). $(C - 1)$ in model M5 is the linear effect of confidence, re-coded so that the intercept indicates the

Parameter	Mammography Estimate	95% CI		Lumbosacral spine Estimate	95% CI	
**M1: Inconsistency (intercept-only model)**						
Intercept	−1.51	−1.75	−1.28	−2.30	−2.72	−1.89
sd(expert)	0.47	0.37	0.58	0.62	0.40	1.06
sd(case)	0.76	0.61	0.98	0.94	0.78	1.13
**M2: Inconsistency vs. case ambiguity (Prediction 1):** $I \sim (\widehat{P} - 0.5) + (\widehat{P} - 0.5)^2$						
Intercept	−1.50	−1.63	−1.37	−2.37	−2.77	−1.96
$(\widehat{P} - 0.5)$	−1.70	−8.45	5.15	−56.75	−63.43	−50.32
$(\widehat{P} - 0.5)^2$	−49.20	−56.82	−42.54	−27.38	−33.38	−21.59
sd(expert)	0.47	0.37	0.59	0.63	0.41	1.06
sd(case)	0.17	0.03	0.30	0.09	0.00	0.27
**M3: Confidence (intercept-only model)**						
Intercept	3.73	3.62	3.84	1.62	1.45	1.80
sd(expert)	0.46	0.40	0.53	0.29	0.20	0.48
sd(case)	0.25	0.21	0.31	0.16	0.15	0.18
**M4: Confidence vs. case ambiguity (Prediction 2):** $C \sim (\widehat{P} - 0.5) + (\widehat{P} - 0.5)^2$						
Intercept	3.73	3.62	3.83	1.62	1.45	1.79
$(\widehat{P} - 0.5)$	2.91	−0.48	6.25	5.45	4.32	6.55
$(\widehat{P} - 0.5)^2$	13.92	10.62	17.20	4.17	3.05	5.32
sd(expert)	0.46	0.40	0.54	0.29	0.20	0.47
sd(case)	0.16	0.13	0.20	0.12	0.11	0.14
**M5: Inconsistency vs. confidence (Prediction 3):** $I \sim (C - 1)$						
Intercept	0.15	−0.21	0.51	−1.37	−1.72	−1.04
(C - 1)	−0.63	−0.74	−0.51	−1.66	−1.92	−1.40
sd(expert)	0.54	0.43	0.66	0.44	0.26	0.77
sd(case)	0.62	0.48	0.81	0.78	0.61	0.96
**M6: Confidence rule vs. first/second diagnoses**						
Intercept	0.74	0.61	0.86	0.45	0.26	0.67
Diagnosis 1	−0.54	−0.71	−0.37	−1.11	−1.37	−0.86
Diagnosis 2	−0.93	−1.10	−0.77	0.22	−0.03	0.47
sd(expert)	0.04	0.00	0.12	0.12	0.01	0.32
sd(case)	0.03	0.00	0.12	0.05	0.00	0.15
**M7: Confidence rule and kind vs. wicked cases (Prediction 4)**						
Intercept	0.90	0.75	1.05	0.53	0.32	0.75
Wicked	−0.57	−0.89	−0.27	−0.35	−1.05	0.40
Diagnosis 1	−0.74	−0.95	−0.55	−1.20	−1.48	−0.91
Diagnosis 1 × Wicked	0.62	0.20	1.07	0.56	−0.44	1.60
Diagnosis 2	−1.05	−1.24	−0.85	0.15	−0.12	0.43
Diagnosis 2 × Wicked	0.51	0.08	0.94	0.13	−0.91	1.17
sd(expert)	0.04	0.00	0.12	0.11	0.01	0.33
sd(case)	0.04	0.00	0.12	0.05	0.00	0.16

Table S1

*Bayesian mixed-level regression models testing predictions 1–4 in the mammography and lumbosacral spine datasets. Posterior distributions of parameters are summarized by their posterior median (*Estimate*) and 95% credible interval (*95% CI*).* sd(expert) *and* sd(case) *show the standard deviations of the group-level distribution (aka "random effects") of the intercept for experts and cases, respectively. See text for more details (incl. coding of variables and interpretation of parameters).*

inconsistency at the lowest confidence level in both datasets (i.e., $C = 1$; because for $C = 1$, zero corresponds to the lowest possible confidence rating because $C - 1 = 0$).

The two models for the confidence rule (M6 and M7) are logistic regression models; their parameters thus indicate changes in log odds. These two models only included those data for which an expert's two diagnoses for the same case differed; all other models use all data. Furthermore, model M7 only considers cases that are clearly kind ($\widehat{P_i} > 0.6$) or clearly wicked ($\widehat{P_i} < 0.4$). This was done to account for the fact that, especially in the spine dataset (with only 13 experts), values for $0.4 < P_i < 0.6$ do not allow for a reliable classification of cases into kind or wicked (see Methods in the main text for more details). The decision of the confidence rule is the reference level; that is, *Diagnosis 1* and *Diagnosis 2* in model M6 indicate the change in accuracy (in log odds) from the confidence rule (*Intercept*) to the first or second diagnosis, respectively. In model M7, kind cases constitute the reference level; that is, *Wicked* indicates for the confidence rule the change in accuracy (in log odds) when considering wicked cases instead of kind cases (the latter represented by *Intercept*). Then *Diagnosis 1* and *Diagnosis 2* indicate for kind cases the change in accuracy (in log odds) when switching from the confidence rule (*Intercept*) to the first or second diagnosis, respectively. The interaction terms (*Diagnosis 1* × *Wicked* and *Diagnosis 2* × *Wicked*) show whether the type of case (kind vs. wicked) moderates the differences between the confidence rule and first and second diagnoses, respectively.

Results

Regression model M2 (Supplementary Table S1) shows a negative quadratic term for case ambiguity in both datasets, supporting the first prediction, that is, the higher a case's ambiguity, the more likely an individual expert will be inconsistent when judging the same case again. Comparing the standard deviations of the group-level intercepts for experts and cases in model M1 (intercept-only model) shows that inconsistency differed more strongly for cases than for experts. Comparing the standard deviations of the group-level intercepts for cases between model M1 (intercept-only) and M2 (incorporating a case's ambiguity $\widehat{P_i}$) shows that the variance among cases is reduced by a factor of 5 in the mammography dataset and by a factor of 11 in the spine dataset—highlighting how much variance in inconsistency can be explained by a case's ambiguity.

Regression model M4 (Supplementary Table S1) shows a positive quadratic term for confidence in both datasets, corroborating the second prediction: the higher a case's ambiguity (indexed by disagreement among experts' initial diagnoses), the less confident an expert will be in their initial decision, irrespective of whether the expert consensus for a case is correct or not. Comparing the standard deviations of the group-level intercepts, the intercept-only model M3 shows that the mean confidence of experts differs more than the mean confidence assigned to those same cases.

Regression model M5 (Supplementary Table S1) shows a negative linear term for confidence for predicting inconsistency in both datasets, corroborating the third prediction: the less confident an expert is in their initial decision, the more likely they will change it when judging on the same case again.

Regression model M6 (Supplementary Table S1) shows a reliably negative coefficient for *Diagnosis 1* in both datasets, indicating that relative to the first diagnosis, choosing the diagnosis with the higher confidence improves accuracy. *Diagnosis 2* was only reliably

negative in the mammography dataset, which indicates that the confidence rule was superior to the second diagnoses only in the mammography, but not the spine dataset—presumably because experts' accuracy improved from the first to the second assessment phase in the latter dataset (see Supplementary Figure S1 and Discussion in the main text). Model M7 (Supplementary Table S1) takes into account the wickedness of cases, and partly supports the fourth prediction: For kind cases, the confidence rule was more accurate than either the first (*Diagnosis 1*) or second diagnosis (*Diagnosis 2*) in the mammography dataset. In the spine dataset, for kind cases the confidence rule was only more accurate than the first diagnosis and similar in accuracy to the second diagnosis. The results for wicked cases were less consistent with our fourth prediction. In both datasets using the confidence rule did not lead to better diagnoses compared with using the first or second diagnoses (see also Figure 3 in the main text).

Additional Results

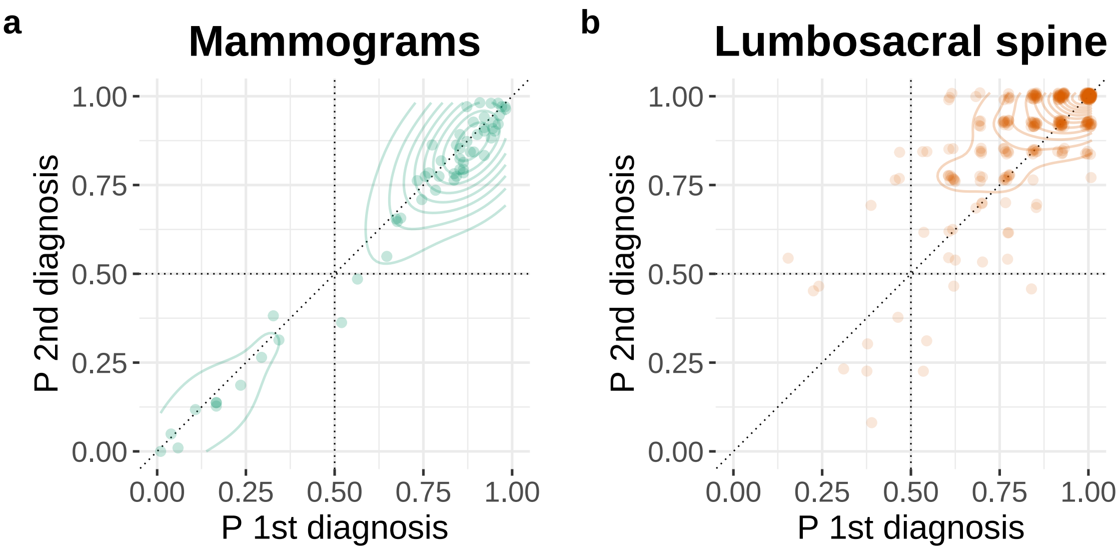

Figure S1. Relation between the proportion of experts who made a correct diagnosis ($\widehat{P}$) in the first vs. second diagnoses across cases. Each dot represents one case. The contour lines show 2D kernel density estimates (using an axis-aligned bivariate normal kernel, evaluated on a square grid) summarizing the bivariate distributions. Panel *b* employs jittering to avoid overplotting. While in the mammograms dataset first and second diagnoses for a case were similarly accurate (panel *a*), in the lumbosacral-spine dataset the second diagnoses were, on average, more accurate than the first ones (panel *b*).

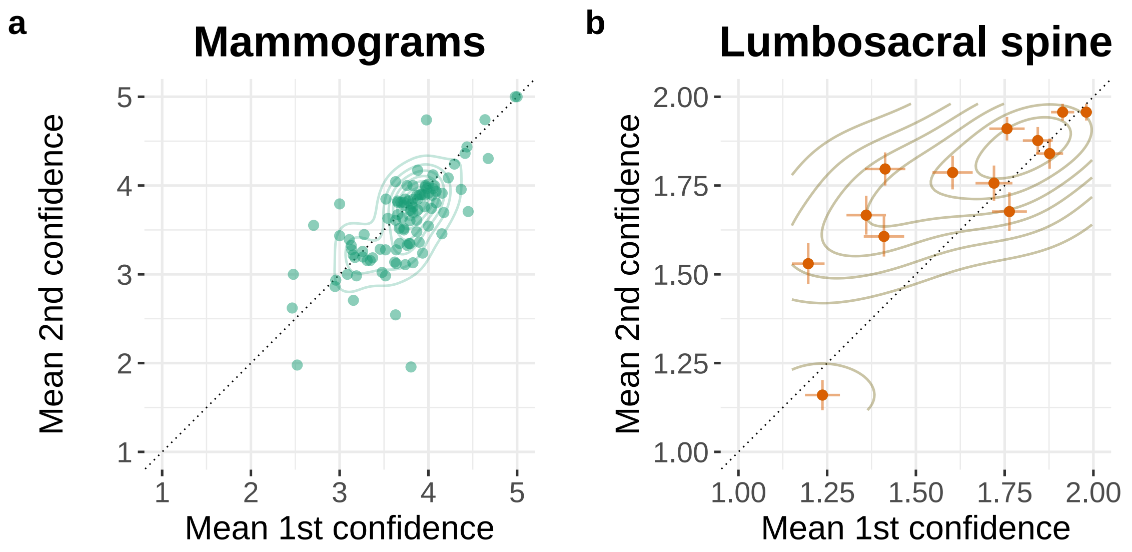

Figure S2. Relation between the mean confidence per expert for first vs. second diagnoses. Each dot represents one expert. In panel *b* (spine dataset), vertical and horizontal error bars per expert are indicated (showing twice the standard error for each expert on either side of the point); in panel *a* (mammography dataset), no such error bars are shown to avoid overplotting. The contour lines show 2D kernel density estimates (using an axis-aligned bivariate normal kernel, evaluated on a square grid) summarizing the bivariate distributions. While in the mammograms dataset experts' first and second diagnoses were rendered with similar confidence (panel *a*), in the lumbosacral-spine dataset the second diagnoses were, on average, rendered with more confidence than the first ones (panel *b*).

References

Bürkner, P.-C. (2017). brms: An R package for Bayesian multilevel models using Stan. *Journal of Statistical Software*, *80*(1), 1–28. doi: 10.18637/jss.v080.i01
